# CAGE-miR-140-5p-Wnt1 Axis Regulates Autophagic Flux, Tumorigenic Potential of Mouse Colon Cancer Cells and Cellular Interactions Mediated by Exosomes

**DOI:** 10.3389/fonc.2019.01240

**Published:** 2019-11-14

**Authors:** Minjeong Yeon, Seungheon Lee, Joo-Eun Lee, Hyun Suk Jung, Youngmi Kim, Dooil Jeoung

**Affiliations:** ^1^Department of Biochemistry, Kangwon National University, Chuncheon-si, South Korea; ^2^College of Medicine, Institute of New Frontier Research, Hallym University, Chuncheon-si, South Korea

**Keywords:** cancer associated gene CAGE, cellular interactions, exosomes, micro RNA-140-5p, tumor microenvironment, wnt1

## Abstract

Although the cancer/testis antigen CAGE has been implicated in tumorigenesis, the molecular mechanisms of CAGE-promoted tumorigenesis remain largely unknown. CT26^Flag−CAGE^ cells, CT26 (mouse colon cancer cells) cells stably expressing CAGE, were established to investigate CAGE-promoted tumorigenesis. Down-regulation of CAGE led to decreased autophagic flux in CT26^Flag−CAGE^ cells. CAGE interacted with Beclin1, a mediator of autophagy. The CT26^Flag−CAGE^ cells showed enhanced autophagosome formation and displayed greater tumor spheroid-forming potential than CT26 cells. MicroRNA array analysis revealed that CAGE decreased the expression of various microRNAs, including miR-140-5p, in CT26 cells. CAGE was shown to bind to the promoter sequences of miR-140-5p. MiR-140-5p inhibition increased the tumorigenic potential of and autophagic flux in CT26 cells. A miR-140-5p mimic exerted negative effects on the tumorigenic potential of CT26^Flag−CAGE^ cells and autophagic flux in CT26^Flag−CAGE^ cells. MiR-140-5p was predicted to bind to the 3′-UTR of Wnt1. CT26^Flag−CAGE^ cells showed higher expression of Wnt1 than CT26 cells. Down-regulation of Wnt1 decreased autophagic flux. Luciferase activity assays showed the direct regulation of wnt1 by miR-140-5p. Tumor tissue derived from the CT26^Flag−CAGE^ cells revealed higher expressions of factors associated with activated mast cells and tumor-associated macrophages than tumor tissue derived from CT26 cells. Culture medium from the CT26^Flag−CAGE^ cells increased autophagic flux in CT26 cells, mast cells and macrophages. Culture medium from the CT26^Flag−CAGE^ cells increased CD163 and autophagic flux in CT26 cells, mast cells, and macrophages in a Wnt1-dependent manner. Exosomes from CT26^Flag−CAGE^ cells increased autophagc flux in CT26 cells, mast cells, and macrophages. Exosomes from CT26^Flag−CAGE^ cells increased the tumorigenic potential of CT26 cells. Wnt1 was shown to be present within the exosomes. Recombinant Wnt1 protein increased autophagic flux in CT26, mast cells, and macrophages. Recombinant wnt1 protein mediated interactions between the CT26 cells, mast cells, and macrophages. Our results showed novel roles for the CAGE-miR-140-5p-Wnt1 axis in autophagic flux and cellular interactions mediated by exosomes.

## Introduction

CAGE, a cancer/testis antigen, is present in the sera of patients with various cancers (1, 2). Furthermore, CAGE predominantly reacts with sera from cancer patients, but not with healthy control (3).

CAGE displays tumor-promoting potential and promotes cell cycle progression by inducing expression of cyclin D1 and E in AP-1 and E2F-dependent manner (4). CAGE stimulates angiogenesis (5, 6) and interacts with HDAC2 and confers resistance to anti-cancer drugs (7). The CAGE-miR-200b negative feedback loop regulates anti-cancer drug-resistance and tumorigenic potential (5).

Phthalate enhances cancer cell metastasis and anti-drug resistance by increasing cancer cell stemness (8). Autophagy promotes cancer stem cell (CSC) characteristics such as self-renewal, tumor initiation, and drug resistance (9). The prosurvival autophagy pathway is critical for CSC maintenance (10). The inhibition of autophagic flux enhances apoptosis and anti-cancer effects in hepatocellular carcinoma cells (11). Autophagic flux is closely related to multiple myeloma stem cell-like properties (12). Cisplatin resistance results from the inhibition of apoptosis and autophagy (13). These reports suggest that CAGE may regulate autophagy and CSC-like properties.

CSCs educate monocytes/macrophages toward tumor associated macrophages (TAMs) and the CSCs and TAMs interact and reciprocally promote stem cell-like properties of CSCs such as self-renewal and anti-cancer drug-resistance (9). TLR2 stimulation of human mast cells promotes the growth of colon cancer spheroids (14). Mast cell-derived mediators activate STAT3 signaling via the down-regulation of GSK3β expression, which in turn inhibits glioma cell proliferation and migration (15). Exosomes from bone marrow stromal macrophages regulate CSC-like properties, by either inducing or reversing dormancy (16). These reports suggest role for exosomes in mediating cellular interactions involving cancer cells and stromal cells within the tumor microenvironment.

We investigated the mechanisms of CAGE-promoted tumorigenesis in detail. We identified miR-140-5p as a direct target of CAGE. We present evidence that the CAGE-miR-140-5p axis regulates autophagic flux, CSC-like properties, and tumorigenic potential. MiR-140-5p acted as a negative regulator of Wnt1. Wnt1 was present within exosomes derived from mouse colon cancer cells expressing CAGE. We present evidence that the CAGE-miR-140-5p-Wnt1 axis regulated cellular interactions within the tumor microenvironment mediated by exosomes. We suggest that CAGE can serve as a target for the development of anti-cancer drugs.

## Materials and Methods

### Materials

An enhanced chemiluminescence (ECL) kit was purchased from Amersham Biosciences. Lipofectamine and Plus™ reagent were purchased from Invitrogen. SiRNAs, miRNA inhibitors, and miRNA mimics were purchased from Bioneer Company (Daejeon, Korea). Goat anti-rabbit IgG (conjugated with HRP) was purchased from Enzo Company (ADI-SAB-300-J), Goat anti-mouse IgG (conjugated with HRP) was purchased from Thermo Fisher Company (31430), and Donkey anti-goat IgG (conjugated with HRP) was purchased from Thermo Fisher Company (A15999). Recombinant wnt1 protein was purchased from R&D systems.

### Cell Lines and Cell Culture

Cancer cell lines used in this study were cultured in Dulbecco's modified minimal essential medium (Invitrogen) supplemented with heat-inactivated 10% fetal bovine serum (Invitrogen) and antibiotics at 37°C in a humidified incubator with a mixture of 95% air and 5% CO_2_. Mouse CT26^Flag−CAGE^ cells that stably express CAGE were established by selection in medium containing G418 (400 μg /ml). CT26^Flag−CAGE^1 cells and CT26^Flag−CAGE^2 cells stably express CAGE. These cells are separate independent clone. CT26 cells were purchased from Korea Cell Line Bank (KCLB 80009). Lung mast cells and lung macrophages were isolated according to standard procedures (17).

### Immunoblot

For PAGE and Western blot, cell or tissues lysates were prepared using lysis buffer (62.5 mm Tris-HCl, pH 6.8, 2% (w/v) SDS, 10% (v/v) glycerol, 50 mm dithiothreitol, 0.01% (w/v) bromphenol blue, 10 mm NaF, 1% (v/v) protease inhibitor mixture, 1 mm sodium orthovanadate). The samples were boiled for 5 min, and equal amounts of protein (20 μg/well) were analyzed on a 10% SDS-PAGE. After electrophoresis, proteins were transferred onto a PVDF membrane (Amersham, Cat.10600023) and subjected to immunoblotting. The membranes are blocked with 2% BSA (Gendepot, Cat.A0100-050) in Tris buffered saline with 0.1% Tween-20 (TBS-T) for 30 min. The membranes were incubated with each primary antibody on a shaker at 4°C overnight. The dilution of each primary antibody was empirically determined. After extensive washing, blots were further incubated with an anti-mouse or anti-rabbit IgG-horseradish peroxidase-conjugated antibody at a 1:3,000 dilution for 1 h at room temperature and were developed using an enhanced chemiluminescence kit (ELPIS, 1073).

### Immunoprecipitation

Cells (1 × 10^7^) were lysed in immunoprecipitation buffer (50 mmol/l HEPES, pH 7.6, 150 mmol/l NaCl, 5 mmol/l EDTA, 0.1% Nonidet P-40). After centrifugation (10 min at 15,000× g) to remove particulate material, the supernatant was incubated with each antibody (2 μg/ml) with constant agitation at 4°C. The immunocomplexes were precipitated with protein A/G-Sepharose (Santa Cruz, sc-2003) and analyzed by Western blot. Two hundred microgram of cell lysates or tissue lysates were subjected to immunoprecipitation.

### Immunofluorescence Staining

Cells were fixed with 4% paraformaldehyde (v/v) and then permeabilized with 0.4% Triton X-100. Cells were incubated with primary antibody specific to LC3 (Cell Signaling, 12741, 1:200), CD163 (AbCam, Ab 182422, 1:100), iNOS (Cell Signaling, 13120, 1:100) or Flag (Sigma, sc-398254, 1:500) for 2 h. Anti-rabbit Alexa Fluor 488 (for detection of LC3 and CD163, 1:500) or anti-goat Alexa Fluor 546 (for detection of iNOS) secondary antibody (Molecular Probes, 1:500) was added to cells and incubated for 1 h. For immunofluorescence staining of exosomes, Exosomes in PBS were applied to fibronectin-coated chamber slides (10 μg/ml) for 24 h at 4°C to allow binding exosomes to the slide surface. Immunofluorescence staining employing anti-TSG101 antibody (Santa Cruz Biotechnology, sc-7964, 1:100) or CD63 (Cusabio, CSB-PA259468, 1:100) was performed as previously described.

### Chemo Invasion and Migration Assays

Invasion and migration potential of cancer cells were determined according to the standard procedures employing transwell chamber system (5, 18). Results were analyzed for statistical significance using the Student's *t-*test. Differences were considered significant when *p* < 0.05.

### MicroRNA Array

MicroRNA array analysis was performed according to the protocols provided by the manufacturer (Koma Biotech).

### RNA Extraction and Quantitative Real Time PCR

Total miRNA was isolated using the *mir*Vana miRNA isolation kit (Ambion). CDNA was synthesized from miRNA with poly (A) tail using a poly (T) adaptor primer and qScript™ reverse transcriptase (Quanta Biogenesis). Expression levels of miR-140-5p was quantified with a SYBR Green qRT-PCR kit (Ambion) using a miRNA-specific forward primer and a universal poly (T) adaptor reverse primer.

### Transfection

Transfections were performed according to the manufacturer's instructions (Invitogen). For miR-140-5p knockdown, cells were transfected with 10 nM oligonucleotide (inhibitor) with Lipofectamine 2000 (Invitrogen), according to the manufacturer's protocol. The sequences used were 5′-CAGUGGUUUUACCCUAUGGUAG-3′ (miR-140-5p inhibitor) and 5′-TAACACGTCTATACGCCCA-3′ (control inhibitor).

### *In vivo* Tumorigenic Potential

Cancer cells (1 × 10^6^) were injected subcutaneously into the dorsal flank area of the BALB/c mice to induce formation of tumors. After tumors reach certain size, control mimic or miR-140-5p mimic (each at 100 nM) was injected five times to determine the effect of miR-140-5p on the tumorigenic potential of CT26^Flag−CAGE^ cells. Control inhibitor or miR-140-5p inhibitor (each at 100 nM) was also injected five times to determine the effect of miR-140-5p on the tumorigenic potential of CT26. To examine whether exosomes would affect the tumorigenic potential, CT26 cells (5 × 10^6^) in 1:1 ratio of exosomes:Matrigel (Growth Factor Reduced; BD Biosciences) were injected subcutaneously in flanks of 8-week-old male nude mice. All animal experiments were performed according to the guide lines of the Korean Council for the Care and Use of Animals in Research and approved by the Institutional Animal Care and Use Committee (IACUC) of Kangwon National University (KIACUC-160329-2).

### Immunohistochemical Staining

The immunohistochemical staining was performed according to the protocols provided by the manufacturer (Vector Laboratories Inc., Burlingame, CA). Tissues were fixed in 10% (v/v) buffered formalin, embedded in paraffin, sectioned at 4–6 μm, Immunohistochemistry staining of tissues was performed by using the avidin-biotin detection method (Vectastain ABC kit, Vector Laboratories Inc., Burlingame, CA). Briefly, 4–6-μm-thick sections of the paraffin-embedded tissue blocks were cut, mounted on positively charged glass slides, and dried in an oven at 56°C for 30 min. The sections were deparaffinized in xylene and then rehydrated in graded ethanol and water. Endogenous peroxidase was blocked by incubation in 3% (v/v) hydrogen peroxide for 15 min. Antigen retrieval was accomplished by pretreatment of the sections with citrate buffer at pH 6.0 for 20 min at 56°C in a microwave oven and then allowing the sections to cool for 30 min at room temperature. Non-specific endogenous protein binding was blocked using 2.5% normal horse serum (Vector, S-2012). The sections were then incubated with primary antibodies overnight at 4°C. The following primary antibodies were used: Flag (Sigma, F31645, 1: 1,000), pAMPK^Thr172^ (R&D Systems, 2535, 1:200), p62 (Santa Cruz, sc-25575, 1:500), tryptase (Santa Cruz, sc-59587,1:100), chymase (Santa Cruz, sc-25575, sc-59586, 1:200), Wnt1 (Abcam, ab-15251, 1:500), β-catenin (Santa Cruz, sc-59737, 1:100), cyclin D1(Santa Cruz, sc-20044, 1:200), or ATG7 (Cell Signaling, 8558. 1:200). After washing, sections were treated with biotinylated secondary antibodies (Vector, MP-7500). The color was developed with diaminobenzidine (Vector, Cat.SK-4100). Sections were counterstained with Mayer's hematoxylin(Dako, S3309).

### Chromatin Immunoprecipitation (ChIP) Assays

ChIP assays were performed according to the standard procedures (19). Mouse miR140-5p promoter-1 [5′-GGTTGTCCTTGGCTACGTG-3′ (forward) and 5′-TAGAAGGAAAGCCAGGGG-3′ (reverse)], miR-140-5p promoter-2 [5′-TATGTGATGCAGCCAGAGCA-3′ (forward) and 5′-CCAGCAAGCAGGGTCAGA-3′ (reverse)] were used.

### Tumor Sphere-Forming Potential Assay

For tumor spheroid forming assay, cells were plated (5 × 10^4^ cells/well) in ultralow attachment plates (Corning Inc.) in DMEM/F12 stem cell medium. Cells were maintained at 37°C in a humidified 5% CO_2_ incubator and fed with 0.2 ml of fresh stem cell medium on days 2, 4, and 6. The total number of spheres was counted after 7 days by inverted microscopy (Olympus).

### Matrigel Plug Assays

Matrigel plug assays employing culture medium were performed according to the standard procedures (5, 20). All animal experiments were performed according to the guide lines of the Korean Council for the Care and Use of Animals in Research and approved by the Institutional Animal Care and Use Committee (IACUC) of Kangwon National University (KIACUC-160329-2).

### Luciferase Activity Assays

A 343-bp mouse wnt1 gene segment encompassing 3′-UTR of wnt1 was PCR-amplified and subcloned into the (XbaI) site of pGL3 luciferase plasmid. The mutant pGL3-3′-UTR-wnt1 construct was made with the QuikChange site-directed mutagenesis kit (Stratagene). Luciferase activity assay was performed according to the standard procedures (19).

### Electron Microscopic Observation of Autophagosomes

Electron microscopic observation of autophagosomes was performed according to the standard procedures (19). Briefly, the cells were dehydrated with a graded acetone series, and embedded into Spurr medium (Electron Microscopy System). The samples were sectioned (60 nm) with an ultra-microtome (RMC MTXL, Arizona, USA), and double-stained with 2% uranyl acetate for 20 min and lead citrate for 10 min. The sections were then viewed under a Tecnai G2 (FEI, USA) TEM at 200 kV.

### Isolation and Characterization of Exosomes

Isolation of the exosomes was performed according to the manufacturer's instructions (System Biosciences, Mountain View, CA). Exosomes were observed under a Tecnai T10 transmission electron microscope (FEI, USA).

### Labeling and Internalization of Exosomes

Exosomes from CT26^Flag−CAGE^1 cells were labeled using PKH67 Fluorescent Cell Linker kits (Sigma-Aldrich, St. Louis, MO). Uptake of exosomes was determined according to the standard procedures (19). Cells were visualized under a confocal laser scanning microscope LX70 FV300 05-LPG-193 (Olympus).

### The Presence of wnt1 in the Exosomes of CT26^**Flag-CAGE**^ Cells

In order to precipitate exosomes, exosomes extractions purified from CT26^Flag−CAGE^ cells were subjected to centrifugation at 60,000 g for 30 min. Precipitated exosomes were collected and cross-linked by 0.1% glutaraldehyde and 2% paraformaldehyde in phosphate buffer (pH 7.4) for 1 h (4°C), then post-fixed in 2% osmium tetroxide for 30 min (4°C). They were dehydrated with a graded series of ethanol and embedded into epoxy resin (PELCO, USA). Ultrathin sections (~80 nm) were prepared from Ultracut UCT (Leica, Germany) and the sections were mounted on copper grids. Followed to the sectioning, it has stained with 1% uranyl acetate for 20 min, and lead citrate for 10 min for the subsequent TEM observations. For immune-gold labeling electron microscopy, ultrathin sections on the grids were treated with 3% sodium (meta) periodate for 30 min for etching and it was treated with 0.02 M glycine (10 min) for quenching the reaction of free aldehyde group. Sections were then washed in deionized water, floated for 1 h in PBS containing 1% BSA. Etching specimens were incubated directly in the primary rabbit or/and mouse antibodies (Anti-Wnt1 or/and Anti-TSG101 antibodies) at 1:20 dilutions for overnight at 4°C. The grid were washed five time with 0.1% BSA in PBS, incubated in secondary antibodies, anti- Rabbit IgG conjugated to 10 nm and anti-mouse IgG conjugated to 25 nm (AURION, Holland) diluted 1:20 in 0.1% BSA-PBS. The sample grids were stained with uranyl acetate and lead citrate. The sectioned and immune-gold labeled grids were examined using a Tecnai T10 (FEI, USA) operated at 100 kV and JEOL-2100F (JEOL, USA) operated at 200 KV.

### Statistical Analysis

Data were analyzed and graphed using GraphPad Prism statistics program (GraphPad Prism software). Results are presented as means ± S.E. Statistical analysis was performed using one way *t*-tests with differences between means considered significant when *p* < 0.05.

## Results

### CAGE Regulates Autophagic Flux and Cancer Stem Cell-Like Properties

**Figure 1:**
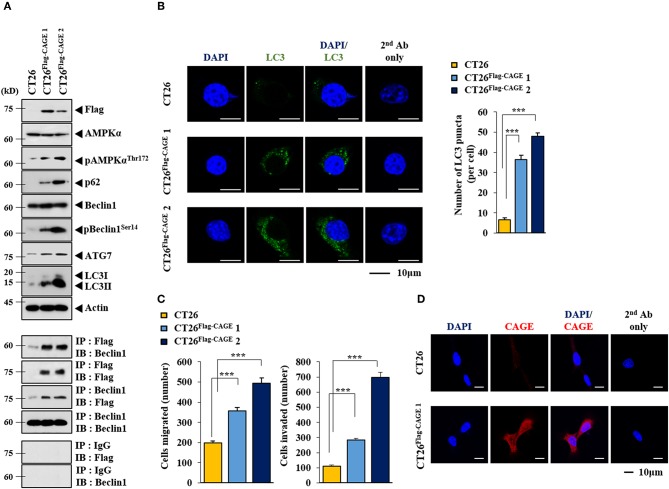
CAGE increases the expression of autophagic flux. **(A)** Cell lysates from the indicated cancer cells were subjected to immunoblot (upper panel) and immunoprecipitation (lower panel). Each blot is a representative of three independent experiments. **(B)** LC3 puncta expression was determined as described. ***p < 0.0005. **(C)** The indicated cancer cells were subjected to migration and invasion assays. ***p < 0.0005. **(D)** Immunofluorescence staining was performed to examine the localization of CAGE.

**Figure 2:**
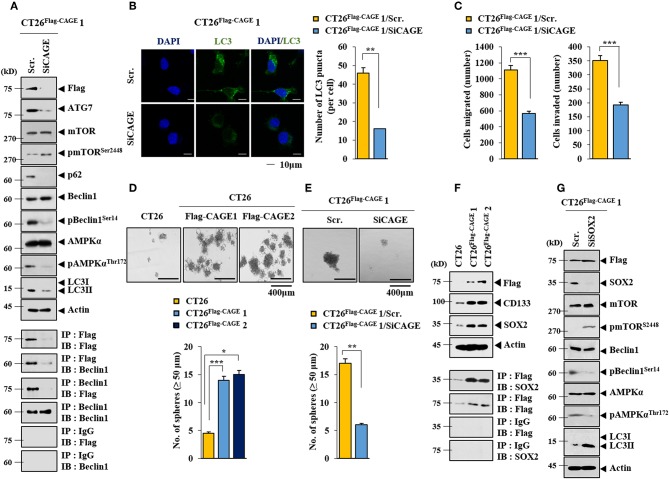
Down-regulation of CAGE decreases autophagic flux, and CSC-like properties. **(A)** The indicated cancer cells were transfected with the indicated siRNA (each at 10 nM) for 48 h, followed by immunoblot (upper) and immunoprecipitation (lower). **(B)** Same as A except that LC3 puncta expression was determined. **p < 0.005. **(C)** The indicated cancer cells were transfected with the indicated siRNA (each at 10 nM) for 48 h, followed by migration and invasion potential assays. ***p < 0.0005. **(D)** Tumor spheroid forming potentials of the indicated cancer cells were determined as described. *p < 0.05; **p < 0.005; ***p < 0.0005. **(E)** Cells were transiently transfected with the indicated siRNA (each at 10 nM) for 48 h. Cells were then subjected to tumor spheroid forming potential assays. **p < 0.005. **(F)** Cell lysates from the indicated cancer cells were subjected to immunoblot (upper) and immunoprecipitation (lower). **(G)** The indicated cancer cells were transiently transfected with the indicated siRNA (each at 10 nM) for 48 h, followed by immunoblot.

Anti-cancer drug-resistance is closely related to autophagy (18, 21). The effect of CAGE on autophagic flux was investigated. For this, we established mouse colon cancer cells stably expressing CAGE. CT26^Flag−CAGE^1 and CT26^Flag−CAGE^2 cells showed higher autophagic flux, such as p62, pBeclin1^Ser14^, LC3II, ATG7, and pAMPKα^T172^ than the parental CT26 cells (Figure 1A). CAGE displayed binding to Beclin1, a mediator of autophagy, in the CT26^Flag−CAGE^1 and CT26^Flag−CAGE^2 cells (Figure 1A). These cells also displayed higher expression of LC3 puncta than the CT26 cells (Figure 1B). The CT26^Flag−CAGE^1 and CT26^Flag−CAGE^2 cells showed higher migration and invasion potential than the parental CT26 cells (Figure 1C). CAGE showed both cytoplasmic and nuclear localization (Figure 1D). CAGE increased autophagosomes formation in the CT26 cells (Supplementary Figure 1). Down-regulation of CAGE led to decreased autophagic flux in the CT26^Flag−CAGE^1 and CT26^Flag−CAGE^2 cells (Figure 2A). Down-regulation of CAGE led to decreased expression of LC3 puncta (Figure 2B) and decreased the migration and invasion potentials of the CT26^Flag−CAGE^1 cells (Figure 2C). Autophagy regulates the expression of pluripotency-associated proteins (PA), such as SOX2, in cervical CSCs (10). Calpain-6 promotes autophagy and maintains the tumor-initiating cell population in sarcoma stem cells (22). We examined the effect of CAGE on CSC-like properties. The CT26^Flag−CAGE^1 and CT26^Flag−CAGE^2 cells showed higher tumor spheroid-forming potential than the CT26 cells (Figure 2D). CAGE was necessary for the tumor spheroid forming potential of CT26^Flag−CAGE^1 cells (Figure 2E). The CT26^Flag−CAGE^1 and CT26^Flag−CAGE^2 cells showed higher expression of CD133 and SOX2, markers of cancer stemness (23), than CT26 (Figure 2F). CAGE interacted with SOX2 (Figure 2F). SOX2 did not affect the expression of CAGE, but decreased autophagic flux in CT26^Flag−CAGE^1 cells (Figure 2G). Thus, CAGE regulates autophagic flux and CSC-like properties.

### CAGE-Beclin1 Interaction Is Necessary for Autophagy

**Figure 3:**
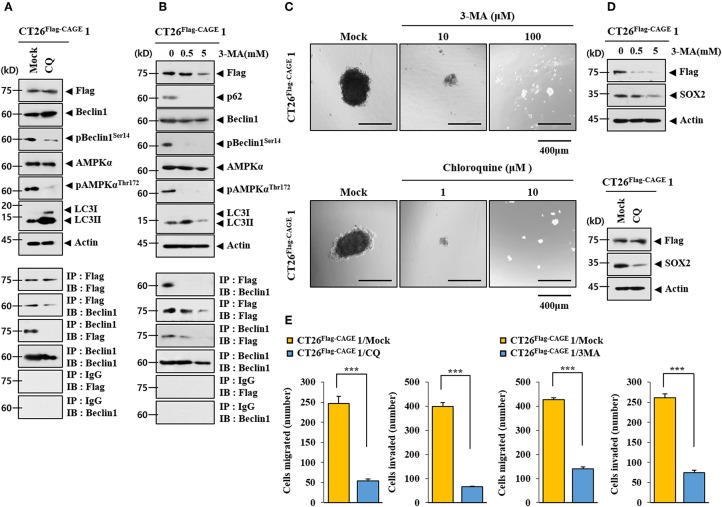
CAGE-Beclin1 interaction is necessary for autophagy. **(A)** The indicated cancer cells were treated with CQ (50 μM) for 12 h, followed by immunoblot and immunoprecipitation. **(B)** The indicated cancer cells were treated with various concentrations of 3-MA for 12 h, followed by immunoblot and immunoprecipitation. **(C)** The indicated cancer cells were treated with CQ or 3-MA at the indicated concentration, followed by tumor spheroid forming potential assays. **(D)** The indicated cancer cells were treated with 3-MA at the indicated concentration for 12 h or treated with CQ (50 μM) for 12 h, followed by immunoblot. **(E)** The indicated cancer cells were treated with CQ (50 μM) for 12 h or with 3-MA (0.5 mM) for 12 h, followed by invasion and migration potential assays. ***p < 0.0005.

CQ (Figure 3A) and 3-MA (Figure 3B), inhibitors of autophagy, decreased autophagic flux and inhibited the interaction between CAGE and Beclin1 in CT26^Flag−CAGE^1 cells. CQ and 3-MA decreased the tumor spheroid-forming potential (Figure 3C) and the expression of SOX2 in CT26^Flag−CAGE^1 cells (Figure 3D). CQ and 3-MA negatively regulated the migration and invasion potential of CT26^Flag−CAGE^1 cells (Figure 3E). These results suggest that CAGE-Beclin1 interaction is necessary for autophagic flux.

### CAGE Enhances the Tumorigenic Potential of CT26

**Figure 4:**
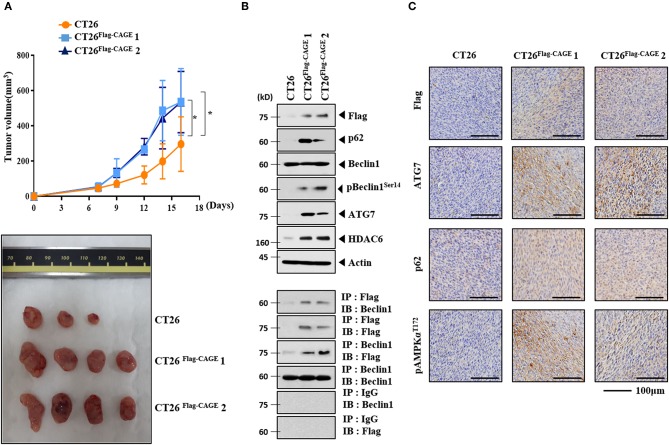
CAGE enhances the tumorigenic potential of CT26 cells. **(A)** The indicated cancer cells (each at 1 × 10^6^) were injected into the dorsal flanks of BALB/C mice. Each value represents an average obtained from BALB/C mice of each group. Data are expressed as a mean ± S.D. Tumor volumes were measured as described. *p < 0.05. Each experimental group consisted of four BALB/C mice. **(B)** Lysates from the indicated tumor tissues were subjected to immunoblot and immunoprecipitation. **(C)** Immunohistochemical staining of tumor tissue employing the indicated antibody (2 μg/ml) was performed. Scale bar represents 100 μm.

CT26^Flag−CAGE^1 and CT26^Flag−CAGE^2 cells showed higher tumorigenic potential than the CT26 cells (Figure 4A). Immunoblots of tumor tissue lysates showed that CAGE increased autophagic flux and demonstrated binding to Beclin1 (Figure 4B). Immunohistochemical staining showed that CAGE increased autophagic flux (Figure 4C). Thus, CAGE enhances the tumorigenic potential of CT26 by increasing autophagic flux.

### Identification of miRNAs Regulated by CAGE

**Figure 5:**
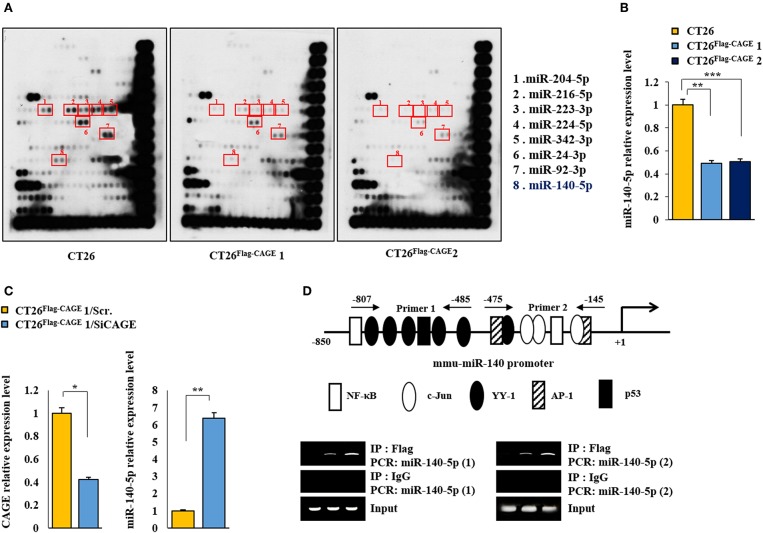
MiR-140-5p serves as a target of CAGE. **(A)** MicroRNA array analysis was performed as described. **(B)** QRT-PCR analysis of the indicated cancer cells was performed. **p < 0.005; ***p < 0.0005. **(C)** CT26 ^Flag−CAGE^1 cells were transfected with the indicated siRNA (each at 10 nM). At 48 h after transfection, cells were subjected to qRT-PCR analysis. *p < 0.05; **p < 0.005. **(D)** Shows potential binding sites for various transcriptional factors in the promoter sequences of miR-140-5p (upper). ChIP assays employing the indicated antibody (2 μg/ml) was performed as described (lower panel).

To identify the miRNAs regulated by CAGE, miRNA array analysis was performed. The CT26^Flag−CAGE^1 and CT26^Flag−CAGE^2 cells showed lower expressions of miRNAs, such as miR-140-5p, than the CT26 cells (Figure 5A). QRT-PCR analysis confirmed the microRNA analysis (Figure 5B). CAGE negatively regulated the expression of miR-140-5p (Figure 5C). The miR-140-5p promoter sequences served as binding sites for transcription factors (Figure 5D, upper). CAGE displayed binding to the promoter sequences of miR-140-5p (Figure 5D, lower). This indicates direct regulation of miR-140-5p by CAGE. Thus, miR-140-5p, a direct target of CAGE, may inhibit autophagic flux.

### MiR-140-5p Regulates Autophagic Flux

**Figure 6:**
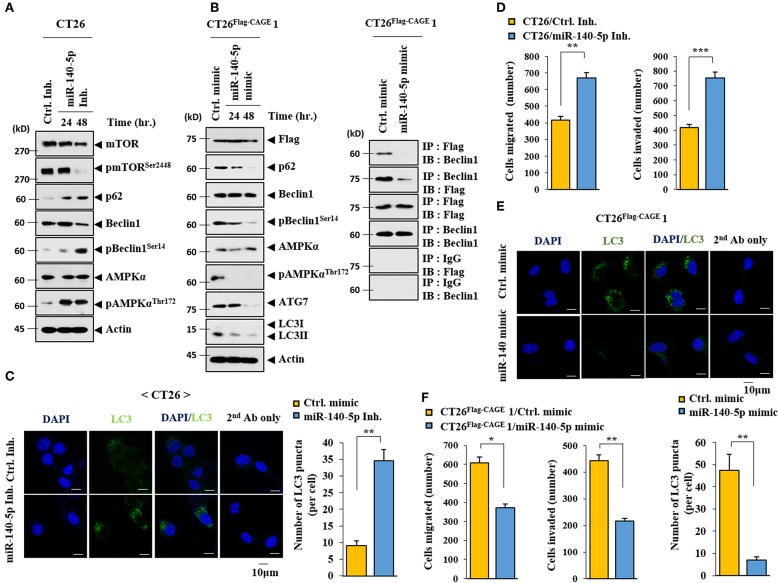
MiR-140-5p regulates autophagic flux. **(A)** CT26 cells were transfected with control inhibitor (10 nM) for 48 h or with miR-140-5p inhibitor (10 nM) for various time intervals. Immunoblot was performed. **(B)** The indicated cancer cells were transfected with control mimic (10 nM) for 48 h or with miR-140-5p mimic (10 nM) for various time intervals, followed by immunoblot (left). The indicated cancer cells were transfected with the indicated mimic (each at 10 nM) for 48 h, followed by immunoprecipitation. **(C)** CT26 cells were transfected with control inhibitor (10 nM) or with miR-140-5p inhibitor (10 nM) for 48 h. LC3 puncta expression was determined. **p < 0.005. **(D)** CT26 cells were transfected with the indicated inhibitor (each at 10 nM) for 48 h, followed by migration or invasion potential assay. **p < 0.005; ***p < 0.0005. **(E)** The indicated cancer cells were transfected with control mimic (10 nM) or with miR-140-5p mimic (10 nM) for 48 h intervals. LC3 puncta expression was determined. **p < 0.005. **(F)** Same as E except that CT26 cells were subjected to migration and invasion assays. *p < 0.05; ***p < 0.005.

The miR-140-5p inhibitor increased autophagic flux while decreasing the expression of pmTOR^Ser2448^, an inhibitor of autophagy, in CT26 cells (Figure 6A). The miR-140-5p mimic exerted the opposite effects on autophagic flux and the interaction between CAGE and Beclin1 in CT26^Flag−CAGE^1 cells (Figure 6B). The miR-140-5p inhibitor increased LC3 puncta expression (Figure 6C) and enhanced the migration and invasion potential of the CT26 cells Figure 6D). The miR-140-5p mimic decreased the expression of LC3 puncta in CT26^Flag−CAGE^1 cells (Figure 6E). MiR-140-5p mimic decreased the migration and invasion potentials of the CT26^Flag−CAGE^1 cells (Figure 6F). Thus, miR-140-5p regulates autophagic fluxes.

### MiR-140-5p Regulates the Tumorigenic Potential of CT26

**Figure 7:**
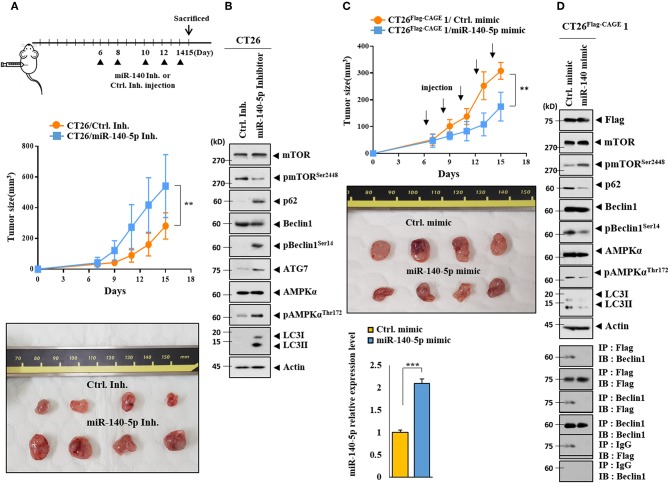
MiR-140-5p regulates the tumorigenic potential. **(A)** CT26 cells (1 × 10^6^) were injected into the dorsal flanks of BALB/C mice. Following the establishment of sizeable tumor, the indicated inhibitor (each at 100 nM) was injected at the indicated day. Each value represents an average obtained from BALB/C mice of each group. Data are expressed as a mean ± S.D. **p < 0.005. Each experimental group consisted of four BALB/C mice. **(B)** Tumor tissue lysates were subjected to immunoblot. **(C)** CT26 ^Flag−CAGE^1 cells (1 × 10^6^) were injected into the dorsal flanks of BALB/C mice. Following the establishment of sizeable tumor, the indicated mimic (each at 100 nM) was intravenously injected five times in a total of 15 days. Tumor tissue lysates were subjected to qRT-PCR analysis (lower). **p < 0.005; ***p < 0.0005. Each experimental group consisted of four BALB/C mice. **(D)** Tumor tissue lysates were subjected to immunoblot (upper) and immunoprecipitation (lower).

The effect of the miR-140-5p inhibitor on the tumorigenic potential of the CT26 cells was examined. The miR-140-5p inhibitor enhanced the tumorigenic potential of the CT26 cells (Figure 7A). MiR-140-5p inhibitor increased autophagic flux while decreasing the expression of pmTOR^Ser2448^ in the CT26 cells (Figure 7B). The miR-140-5p mimic decreased the tumorigenic potential of the CT26^Flag−CAGE^1 cells (Figure 7C). QRT-PCR showed the expression of miR-140-5p in tumor tissue lysates (Figure 7C). The miR-140-5p mimic decreased autophagic flux and inhibited the interaction between CAGE and Beclin1 (Figure 7D). Thus, miR-140-5p regulates tumorigenic potential and autophagic flux.

### MiR-140-5p Directly Targets Wnt1

**Figure 8:**
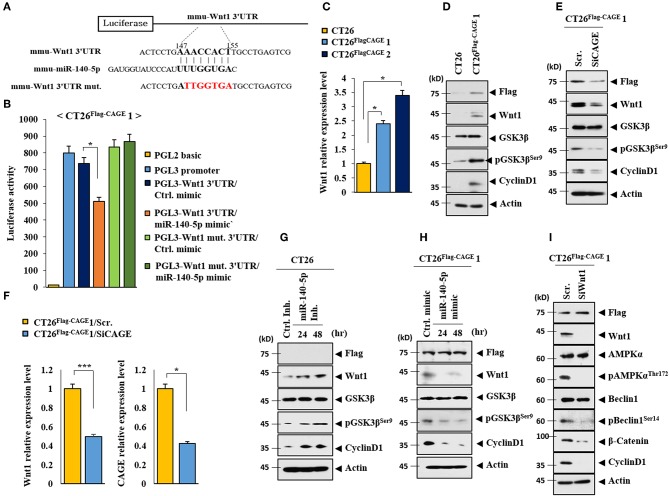
CAGE and miR-140-5p regulate the expression of wnt1. **(A)** Potential binding of miR-140-5p to 3′-UTR of wnt1. **(B)** Wild type Luc-p62-3′-UTR or mutant Luc-p62-3′-UTR was transfected along with control mimic or miR-135-5p mimic (each at 10 nM) into the indicated cell line. At 48 h after transfection, luciferase activity assays were performed. *p < 0.05. **(C)** Cell lysates from the indicated cancer cells were subjected to qRT-PCR analysis. *p < 0.05. **(D)** Cell lysates from the indicated cancer cells were subjected to immunoblot. **(E)** The indicated cancer cells were transfected with the indicated siRNA (each at 10 nM) for 48 h, followed by immunoblot. **(F)** Same as E except that qRT-PCR analysis was performed. *p < 0.05; ***p < 0.0005. **(G)** The indicated cancer cells were transfected with control inhibitor (10 nM) for 48 h or miR-140-5p inhibitor (10 nM) for various time intervals. **(H)** The indicated cancer cells were transfected with control mimic (10 nM) for 48 h or miR-140-5p mimic (10 nM) for various time intervals, followed by immunoblot. **(I)** The indicated cancer cells were transfected with the indicated siRNA (each at 10 nM) for 48 h, followed by immunoblot analysis.

TargetScan analysis predicted that Wnt1 would be a target of miR-140-5p (Figure 8A). It is reasonable that miR-140-5p may directly regulate Wnt1 expression level. Both the wild type and mutant 3′-UTR of Wnt1 showed luciferase activities in CT26^Flag−CAGE^1 cells (Figure 8B). The miR-140-5p mimic inhibited the luciferase activity associated with the Luc-3′-wild type UTR of wnt1, but not the luciferase activity associated with the Luc-3′-mutant UTR of Wnt1 (Figure 8B). Wnt 1 may act as a direct target of miR-140-5p to regulate autophagic flux. CAGE increases the expression of cyclinD1 in an AP1-dependent manner (4). Prodigiosin decreases the tumorigenic potential and expression of cyclin D1 in breast cancer cells by inhibiting the Wnt/β-catenin pathway (24). The CT26^Flag−CAGE^1 cells and CT26^Flag−CAGE^2 cells showed higher expression of Wnt1 mRNA than the CT26 cells (Figure 8C). CT26^Flag−CAGE^1 cells showed higher expressions of Wnt1, pGSK3β^Ser9^ (an inactive form of GSK3β), and cyclinD1 than the CT26 cells (Figure 8D). Inhibition of Wnt1 prevents leptin-stimulated GSK3β phosphorylation (25). Down-regulation of CAGE led to the decreased expression of Wnt1, pGSK3β^Ser9^, and cyclinD1 in the CT26^Flag−CAGE^1 cells (Figure 8E). CAGE also regulated the expression of Wnt1 at the transcription level (Figure 8F). The miR-140-5p inhibitor increased the expression of Wnt1, pGSK3β^Ser9^, and cyclinD1 in the CT26 cells (Figure 8G) while the miR-140-5p mimic exerted the opposite effect on the expression of these proteins in the CT26^Flag−CAGE^1 cells (Figure 8H). The down-regulation of Wnt1 decreased autophagic flux, pGSK3β^Ser9^, and cyclinD1 in the CT26^Flag−CAGE^1 cells (Figure 8I). Tumor tissue lysates of the CT26^Flag−CAGE^1 cells showed higher expression of Wnt1, β-catenin, pGSK3β^Ser9^, and cyclin D1 than the tumor tissue lysates of the CT26 cells (Supplementary Figure 2A). The autophagic degradation of β-catenin by p62 led to a decreased self-renewal capacity of the colonospheres (26). Immunohistochemical staining showed the same results as the immunoblot (Supplementary Figure 2B). Thus, CAGE and miR-140-5p exerted opposite effects on the expression of Wnt1 to regulate autophagic flux.

### CAGE May Mediate Cellular Interactions Within the Tumor Microenvironment

**Figure 9:**
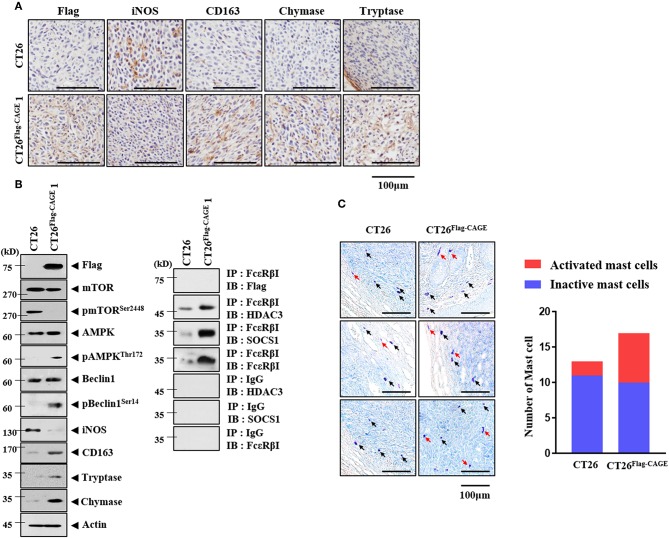
CAGE may promote activation of macrophages and mast cells. **(A)** Tumor tissues were subjected to immunohistochemical staining as described. **(B)** Tumor tissue lysates were subjected to immunoblot (left) and immunoprecipitation (right). **(C)** Tumor tissues were subjected to toluidine blue staining. Red arrows represent activated mast cells. Black arrows represent mast cells.

Cancer cells interact with various stromal cells, such as mast cells, within the tumor microenvironment (27). Thus, we examined whether CAGE would affect cellular interactions within the tumor microenvironment. Tumor tissue derived from CT26^Flag−CAGE^1 cells showed higher expression of CD163, tryptase, and chymase but lower expression of iNOS than the tumor tissue derived from the CT26 cells (Figure 9A). CD163 and iNOS are makers of TAM and M1 macrophages, respectively. Tryptase and chymase are hallmarks of allergic inflammation. Tumor tissue lysates of the CT26^Flag−CAGE^1 cells showed higher expression of autophagic flux, tryptase, chymase, and CD163 but lower expression of iNOS than CT26 tumor tissue lysates (Figure 9B). Tumor tissue lysates of the CT26^Flag−CAGE^1 cells showed the interaction of FcϵRIβ with HDAC3 and SOCS1 (Figure 9B). Tumor tissue lysates of the CT26^Flag−CAGE^1 cells showed more activated mast cells than the tumor tissue lysates of CT26 cells (Figure 9C). Thus, CAGE may mediate cellular interactions within the tumor microenvironment.

### Exosomes Mediate Cellular Interactions

**Figure 10:**
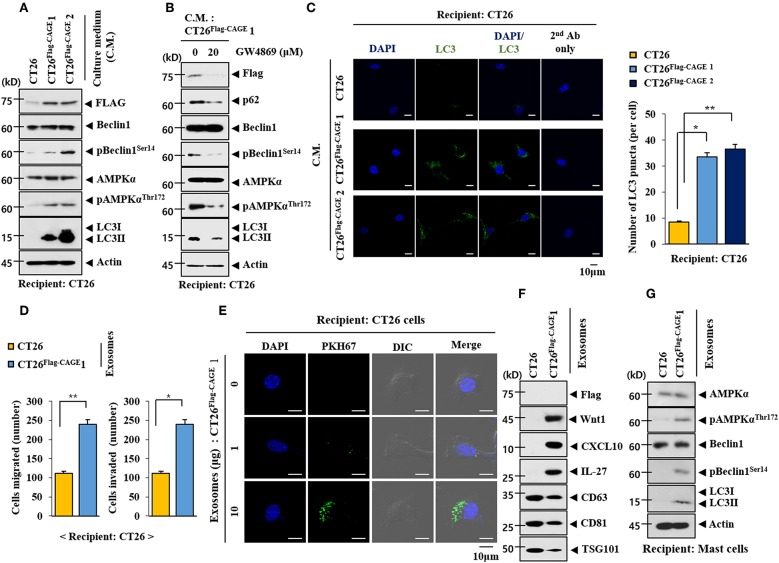
Exosomes of CT26^Flag−CAGE^1 cells increase autophagic flux. **(A)** Culture medium of the indicated cancer cells was added to CT26 cells for 24 h, followed by immunoblot. **(B)** CT26^Flag−CAGE^1 cells were treated without or with GW4869 (10 μM) for 24 h. Culture medium was then added to CT26 cells for 24 h, followed by immunoblot. **(C)** Same as A except that LC3 puncta expression was determined. *p < 0.05; **p < 0.005. **(D)** Exosomes (10 μg) from the indicated cancer cells were added to CT26 cells for 24 h, followed by invasion and migration potential assays. *p < 0.05; **p < 0.005. **(E)** Exosomes (10 μg) from CT26^Flag−CAGE^1 cells were labeled with PKH67 and added to CT26 for 24 h. Cells were visualized under a confocal laser scanning microscope. **(F)** Exosomes (10 μg) from the indicated cancer cells were subjected to immunoblot. **(G)** Exosomes (10 μg) from the indicated cancer cells were added to lung mast cells for 24 h, followed by immunoblot.

We examined whether CAGE would mediate cellular interactions. Culture medium from the CT26^Flag−CAGE^1 and CT26^Flag−CAGE^2 cells increased autophagic flux in the CT26 cells (Figure 10A). Culture medium from the CT26^Flag−CAGE^1 cells treated with GW4869, an inhibitor of exosomes formation, did not increase autophagic flux in the CT26 cells (Figure 10B). It is probable that exosomes from the CT26^Flag−CAGE^1 cells may have increased autophagic flux in the CT26 cells. Culture medium from the CT26^Flag−CAGE^1 cells increased LC3 puncta expression in the CT26 cells (Figure 10C). Immunofluorescence staining of the exosomes showed the co-localization of Wnt1 with TSG101, an exosomal marker (Supplementary Figure 3A). Wnt1 was detected in the lumen of the exosomes, whereas TSG101, a known membrane marker of exosomes, was detected in the outer membrane of the exosomes based on immunogold staining of Wnt1 (as shown by 10 nm golds located in the inner of the vesicles) and TSG101 (as shown by 25 nm golds located in the outer membrane of the vesicles) (Supplementary Figure 3B). Exosomes from the CT26^Flag−CAGE^1 cells enhanced the migration and invasion potential of the CT26 cells (Figure 10D). Immunofluorescence staining showed that the PKH67-labeled exososmes from CT26^Flag−CAGE^1 cells could be transferred to the CT26 cells (Figure 10E). Immunoblot showed the expression of Wnt1 within exosomes from the CT26^Flag−CAGE^1 cells (Figure 10F). Exosomes from the CT26^Flag−CAGE^1 cells also showed the presence of CXCL10 and IL-27 (Figure 10F). Rapamycin, an inducer of autophagy, enhanced the cytotoxic effect of NK cells by increasing the expression of IL-27 in uterine endometrial cancer cells (28). Exosomes from the CT26^Flag−CAGE^1 cells also increased autophagic flux in lung mast cells (Figure 10G). It is probable that the exosomes mediate the effects of CAGE on autophagic flux and cellular interactions. These results suggest that Wnt1 may mediate cellular interactions within the tumor microenvironment.

### Exosomes From CT26^**Flag-CAGE**^ Cells Activate Macrophages

**Figure 11:**
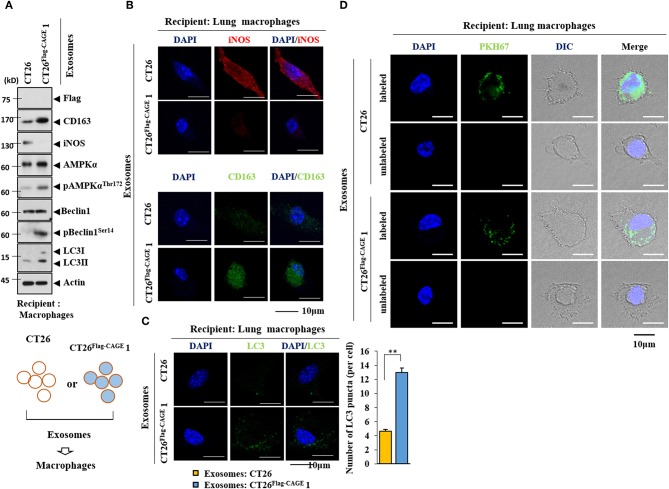
Exosomes mediate the effect of CAGE on the activation of macrophages. **(A)** Exosomes (10 μg) from the indicated cancer cells were added to lung macrophages for 24 h, followed by immunoblot. **(B,C)** Same as A except that immunofluorescence staining employing the indicated antibodies (2 μg/ml) was performed. **p < 0.005. **(D)** Exosomes (10 μg) from the indicated cancer cells were labeled with PKH67 and added to lung macrophages for 24 h. Cells were visualized under a confocal laser scanning microscope.

Exosomes form the CT26^Flag−CAGE^1 cells increased the expression of CD163, but decreased the expression of iNOS in lung macrophages (Figure 11A). Exosomes from the CT26^FlagG−CAGE^ 1 cells also increased the expression of autophagic flux in macrophages (Figure 11A). Immunofluorescence staining showed that exosomes from the CT26^Flag−CAGE^1 cells increased the expression of CD163 (Figure 11B) and LC3 (Figure 11C) and decreased the expression of iNOS (Figure 11B). PKH67-labedled exosomes from the CT26^Flag−CAGE^1 cells were transferred to the lung macrophages (Figure 11D). Thus, exosomes mediated the activation of macrophages by CT26^Flag−CAGE^1 cells.

### Exosomes From CT26^**Flag-CAGE**^ Cells Enhance the Tumorigenic Potential of CT26 Cells

**Figure 12:**
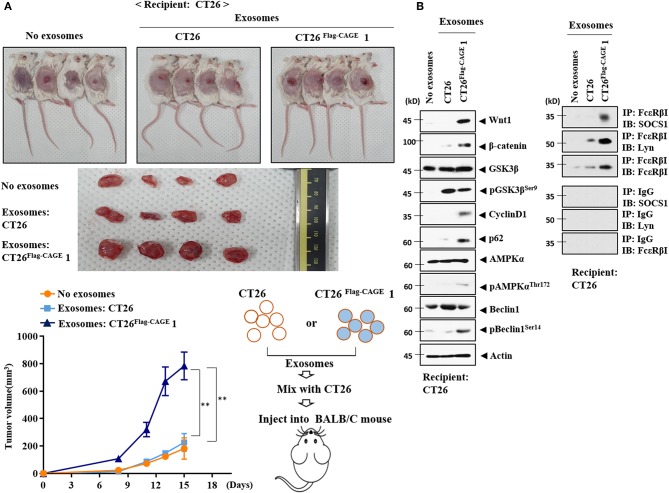
Exosomes of CT26^Flag−CAGE^1 cells enhance the tumorigenic potential of CT26 cells. **(A)** CT26 cells (1 × 10^6^) mixed without or with the exosomes (10 μg) of the indicated cancer cells were injected into the dorsal flanks of BALB/C mice. Each value represents an average obtained from BALB/C mice of each group. Data are expressed as a mean ± S.D. Tumor volumes were measured as described. Each experimental group consisted of four BALB/C mice. **(B)** Lysates from the indicated tumor tissues were subjected to immunoblot and immunoprecipitation.

Exosomes from the CT26^Flag−CAGE^1 cells enhanced the tumorigenic potential of CT26 cells (Figure 12A), but those of the CT26 cells did not affect the tumorigenic potential of CT26. Tumor tissue lysates of CT26 cells that received exosomes from the CT26^Flag−CAGE^1 cells displayed higher expressions of Wnt1, β-catenin, cycinD1, pGSK3β^Ser9^, and autophagic flux than tumor tissue lysates from the CT26 cells that received CT26 exosomes (Figure 12B). Tumor tissue lysates from CT26 cells that received exosomes from the CT26^Flag−CAGE^1 cells displayed increased FcεRIβ expression, and interactions of FcεRIβ with SOCS1 and Lyn (Figure 12B) compared to the tumor tissue lysates of CT26 cells that received CT26 exosomes. Immunohistochemical staining confirmed the immunoblot results (Supplementary Figure 4). The CT26 tumor that received exosomes from the CT26^Flag−CAGE^1 cells showed more activated mast cells than the CT26 tumor tissue that received CT26 exosomes (Supplementary Figure 4). Matrigel plug assays employing culture medium showed that Wnt1 was necessary for the angiogenic potential of the CT26^Flag−CAGE^1 cells (Supplementary Figure 5). MiR-140-5p negatively regulated the angiogenic potential of CT26^Flag−CAGE^1 cells (Supplementary Figure 5). This suggests that the culture medium of CT26^Flag−CAGE^1 cells may promote the tumorigenic potential of CT26 cells. Thus, the exosomes from the CT26^Flag−CAGE^1 cells enhanced the tumorigenic potential of the CT26 cells by increasing autophagic flux and promoting cellular interactions.

### MiR-140-5p-Wnt 1 Regulates Cellular Interactions

**Figure 13:**
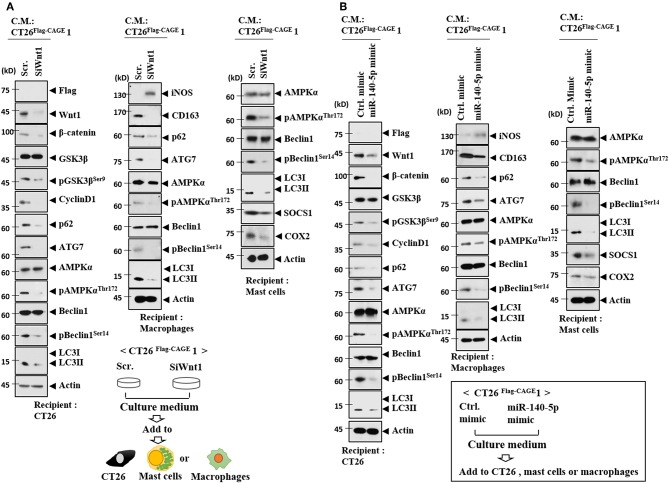
MiR-140-5p and wnt1 regulate cellular interactions. **(A)** The indicated cancer cells were transfected with the indicated siRNA (each at 10 nM) for 48 h. Culture medium was added to CT26 cells, mast cells or lung macrophages for 24 h, followed by immunoblot. **(B)** The indicated cancer cells were transfected with the indicated mimic (each at 10 nM) for 48 h. Culture medium was added to CT26 cells or lung macrophages for 24 h, followed by immunoblot.

Wnt1 was necessary for the increased autophagic flux, Wnt1, β-catenin, and cyclin D1 in CT26 cells induced by the culture medium from CT26^Flag−CAGE^1 cells (Figure 13A). Wnt1 was necessary for the increased autophagic flux and CD163 in lung macrophages activated by the culture medium from the CT26^Flag−CAGE^1 cells (Figure 13A). Down-regulation of Wnt1 exerted a negative effect on the increased autophagic flux, and SOCS1, and COX2 in lung mast cell activated by the culture medium from the CT26^Flag−CAGE^1 cells (Figure 13A). MiR-140-5p prevented the CT26^Flag−CAGE^1 cell culture medium from increasing autophagic flux in the CT26 cells (Figure 13B). The overexpression of miR-140-5p prevented culture medium from the CT26^Flag−CAGE^1 cells from increasing autophagic flux and CD163 in lung macrophages (Figure 13B). MiR-140-5p exerted negative effects on the increased autophagic flux and hallmarks of allergic inflammation in lung mast cells activated by CT26^Flag−CAGE^1 cell culture medium (Figure 13B). Immunofluorescence staining showed that Wnt1 was necessary for the effects of CT26^Flag−CAGE^1 cell culture medium on the expression of CD163, LC3, and iNOS in macrophages (Supplementary Figure 6A). MiR-140-5p mimic or Wnt1 siRNA prevented the CT26^Flag−CAGE^1 cell culture medium from regulating the expression of CD163, LC3, and iNOS in lung macrophages (Supplementary Figure 6B). Thus, the miR-140-5p-Wnt1 axis regulates cellular interactions.

### Wnt1 Mediates Cellular Interactions

**Figure 14:**
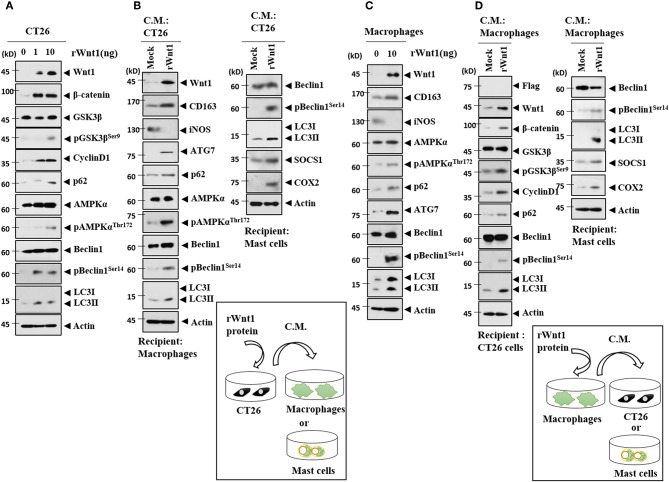
Wnt1 mediates cellular interactions. **(A)** Recombinant wnt1 protein at the indicated concentration was added to CT26 cells for 24 h, followed by immunoblot. **(B)** CT26 cells were treated with recombinant wnt1 protein (10 ng/ml) for 24 h. Culture medium was then added to lung macrophages or lung mast cells for 24 h, followed by immunoblot. **(C)** Recombinant wnt1 protein (10 ng/ml) was added to macrophages for 24 h, followed by immunoblot. **(D)** Macrophages were treated with recombinant wnt1 protein (10 ng/ml) for 24 h. Culture medium was then added to CT26 cells for 24 h, followed by immunoblot.

Because Wnt1 was present within the exosomes from the CT26^Flag−CAGE^1 cells (Figure 10D), the direct effects of Wnt1 on cellular interactions were investigated. Recombinant Wnt1 protein (rWnt1) increased autophagic flux, Wnt1, β-catenin, and cyclin D1 in CT26 cells (Figure 14A). Culture medium from CT26 cells treated with rWnt1 increased autophagic flux and CD163, but decreased iNOS expression in macrophages (Figure 14B). Culture medium from CT26 cells treated with rWnt1 increased autophagic flux, SOCS1, and COX2 in lung mast cells (Figure 14B). Recombinant Wnt1 protein increased autophagic flux and CD163, but decreased the expression of iNOS in macrophages (Figure 14C). Culture medium from macrophages treated with rWnt1 increased autophagic flux, Wnt1, β-catenin, and cyclin D1 in CT26 cells (Figure 14D). Culture medium of macrophages treated with rWnt1 increased autophagic flux, SOCS1, and COX2 in mast cells (Figure 14D). Immunofluorescence staining showed that culture medium from CT26 cells treated with rWnt1 protein increased the expression of CD163 and LC3, but decreased the expression of iNOS in macrophages (Supplementary Figure 7A). Recombinant Wnt1 protein increased the expression of CD163 and LC3, but decreased the expression of iNOS in macrophages (Supplementary Figure 7B). This suggests that Wnt1 may directly mediate cellular interactions.

In summary, we have shown that the CAGE-miR-140-5p-Wnt1 axis regulates autophagic flux, CSC-like properties, and tumorigenic potential. By employing culture medium, we demonstrated interactions between CT26^Flag−CAGE^ cells, mast cells, and macrophages. We also show that exosomes containing Wnt1 mediated these cellular interactions.

## Discussion

EGFR signaling is necessary for the initiation and progression of the autophagic process (29). The decreased expression of HER2 by the downregualtion of Beclin1 confers sensitivity to anti-cancer drugs such as tamoxifen (30). CAGE interacts with EGFR and HER2 in human melanoma cells (31). We, therefore, hypothesized that CAGE would regulate autophagic flux. We found that the overexpression of CAGE in CT26 cells increased autophagic flux and induced an interaction between CAGE and Beclin1. The overexpression of CAGE in CT26 cells also enhanced the formation of autophagosomes. These results suggest the role of CAGE in autophagy. The identification of the CAGE domain necessary for Beclin1 interaction will provide valuable information for the development of CAGE-targeting anti-cancer drugs.

The phosphorylation of ULK1 by AMPK, a mediator of autophagy, is essential for self-renewal and pluripotency in embryonal stem cells (32). Autophagy-related gene 7 (ATG7) induces the binding of β-catenin to the promoter sequences of OCT4 to increase the expression of OCT4, which promotes self-renewal, tumor initiation, and drug resistance (9). Inhibition of NANOG decreases autophagy in tumor cells (9). We showed that the inhibition of autophagy negatively regulated CSC-like properties. CAGE increased the expression of markers of cancer stemness, such as, SOX2, and showed an interaction with SOX2 (33). Identification of the CAGE domain necessary for SOX2 interaction is necessary to better understand CAGE-prompted CSC-like properties. HDAC6 regulates the expression of pluripotency factors, such as POU5F1, NANOG, and SOX2, and is necessary for the pluripotency of CSCs (34). P62 binds to HDAC6 and regulates the deacetylase activity of HDAC6 (35). Thus, it is reasonable that HDAC6 may mediate the effects of CAGE on autophagic flux and tumorigenesis.

MicroRNA array analysis revealed that CAGE decreased the expression of miR-140-5p. MiR-140-5p/miR-140-3p-null mouse shows an increased number of Leydig cells in the developing XY gonad (36). This suggests a regulatory role for miR-140-5p/miR-140-3p in testis differentiation. CAGE, like other cancer/testis antigens, is assumed to be involved in testis development. ChIP assays revealed the direct regulation of miR-140-5p by CAGE. Vitamin D receptor increases the expression of miR-140-5p, which in turn inhibits MAPK signaling in osteoblasts (37). It is reasonable that CAGE may affect MAPK signaling in CT26.

MiR-140 regulates the parathyroid hormone (PTH)-related peptide (PTHrP)-HDAC4 pathway to control chondrocyte differentiation (38). HDAC4 is necessary for autophagy and vascular inflammation through its effect on FoxO3a (39). HDAC4 promotes autophagy and anti-apoptosis and confers resistance to cisplatin (13). Therefore, it would be interesting to examine the effect of CAGE on the expression of HDAC4.

We showed the binding of miR-140-5p to the 3′UTR of Wnt1. SOX2 regulates the expression of Wnt1 in lung cancer cells (40). Wnt1 promotes mammary tumorigenesis (41) and CSC activity by increasing mitochondrial mass (42). Downregualtion of Wnt1 inhibits the growth of hepatic cancer cells by inducing cellular apoptosis (43). Luciferase activity assays showed the direct regulation of Wnt1by miR-140-5p. We showed that CAGE increased the expression of Wnt1 in CT26 cells and the downregulation of Wnt1 led to decreased autophagic flux in CT26^Flag−CAGE^1 cells. Wnt1-inducible signaling pathway protein-3 (WISP-3, also termed CCN6) regulates cell proliferation, differentiation, and migration (44). It is probable that CAGE may regulate the expression of WISP-3.

MiRNA array analysis revealed that CAGE decreased the expression of miR-24-3p. MiR-24-3p was predicted to be a negative regulator of TCF7. TCF7 activates the WNT/β-catenin signaling pathway (45). MiR-24-3p and miR-92-3p, decreased by CAGE, were predicted to target WNT8B. It would be interesting to examine the effects of these miRNAs on autophagic flux in CT26^Flag−CAGE^ cells. MiR-342-3p, decreased by CAGE, was predicted to target RICTOR. RICTOR promotes autophagy and tumor angiogenesis (46). MiR-216-5p, decreased by CAGE, was predicted to target ATG12. The knockdown of ATG12 impairs the effects of miR-1265 inhibition on gastric cancer progression and oncogenic autophagy (47).

Tumor-stromal interaction is critical for the progression of cancers (27). The conditioned medium of human mast cells increased anti-cancer drug-resistance by reducing apoptosis in human pancreatic ductal adenocarcinoma cells (27). Pancreatic cancer cells have been shown to induce mast cell migration and the culture medium of mast cells enhanced cancer cell invasion and proliferation (48). Mast cells were reported to accumulate in colorectal cancer tissues and their density was correlated with cancer progression. The interaction between mast cells and human colon cancer cells is mediated by CCL15 or SCF (14). Mast cells promote colon cancer cell growth by inducing the production of multiple cytokines from cancer cells (14). The tumor tissue lysates of CT26^Flag−CAGE^1 cells revealed the activation of mast cells based on the induction of interactions of FcεRIβ with HDAC3 and SOCS1.

The tumor tissue lysates of CT26^Flag−CAGE^1 cells revealed the activated macrophages based on the higher expression of CD163 than in CT26 tumor lysates. This suggests interactions between CT26^Flag−CAGE^1 cells and macrophages. Tumor-infiltrating macrophages promote glioma cell survival and stimulate angiogenesis by secreted phospho protein 1 (SPP1) (49). Alternatively activated macrophages (AAM)-derived factors utilize a JAK/STAT signaling pathway to induce ovarian cancer metastasis (50). IL-32γ has been known to mediate the effect of multiple myeloma cells on enhancing immunosuppressive function of macrophages (51). These reports suggest cancer cell-macrophage interactions lead to tumor growth.

The inhibition of phosphoinositide kinase PIKfyve increases the secretion of exosomes containing autophagy-related proteins and induces autophagy (52). Exosomes from the gefitinib-treated PC9 cells (Exo-GF) increase autophagic activity and confer resistance to cisplatin (53). Exosomes from pigment epithelium-derived factor (PEDF)-modified adipocyte-derived stem cells (ADSCs) attenuate cerebral injury by activating autophagy and modulating apoptosis (9). Thus, exosomes mediate cellular interactions by regulating autophagy.

Exosomes from AsPC-1, an ascites-derived human pancreatic ductal carcinoma (PDAC) cell line, increase the levels of M2 macrophages markers, such as CD163 (54). Macrophages treated with AsPC-1 exosomes increased the secretion of pro-tumoral, bioactive molecules including VEGF, MCP-1, IL-6, and MMP-9 (54). Exosomes from human mast cells activate KIT-SCF signal transduction and accelerate the proliferation of human lung adenocarcinoma cells (55). Exosomes from miR181-5p-adipose-derived stem cells (ADSCs) activate autophagy by decreasing the expression of Stat3 and Bcl-2 in mouse hepatic stellate (HST-T6) cells (56). These reports suggest that exosomes may mediate cellular interactions. We found that exosomes from CT26^Flag−CAGE^1 cells increased autophagic flux in mast cells and macrophages. Identification of exosomal cytokines and miRNAs that mediate these cellular interactions will facilitates the understanding of tumor-stroma interactions within the tumor microenvironment.

M2 macrophages, but not M1 macrophages, activate the Wnt signaling pathway in epithelial cells (57). IL-10 derived from macrophages activates cAMP response element-binding protein (CREB) and induces the secretion of the pro-repair WNT1-inducible signaling protein 1 (WISP-1) (58). These reports suggest that Wnt1 may mediate cellular interactions. We showed the presence of Wnt1 protein in the exosomes of CT26^Flag−CAGE^1 cells. Culture medium from CT26 cells treated with recombinant Wnt1 protein increased the expression of CD163 in lung macrophages. We showed that recombinant Wnt1 protein increased the expression of CD163 in lung macrophages. Culture medium of macrophages treated with recombinant wnt1 protein increased the expression of autophagic flux in CT26 cells. It is, therefore, probable that Wnt1 mediates cellular interactions within the tumor microenvironment. Exosomal Wnt1 protein enhances the proliferation and migration of colorectal cancer by activating non-canonicial Wnt signaling (59). Identification of cytokines and miRNAs that are regulated by exosomes is necessary for understanding of exosomal-mediated cellular interactions.

CAGE acts as an upstream direct regulator of miR-140-5p and enhances autophagic flux and tumorigenic potential. MiR-140-5p negatively regulates the expression of Wnt1, autophagic flux and tumorigenic potential. Tumor tissue derived from CAGE-expressing cancer cells shows the activation of mast cells and macrophages. We presented evidence that CAGE mediated interactions between cancer cells, mast cells, and macrophages. Wnt1 is present within the exosomes of CAGE-expressing cancer cells and we show that exosomes and Wnt1 mediate these cellular interactions. Our results suggest CAGE as a target for the development of anti-cancer drugs.

## Data Availability Statement

All datasets generated for this study are included in the article/Supplementary Material.

## Ethics Statement

The animal study was reviewed and approved by Institutional Animal Care and Use Committee (IACUC) of Kangwon National University (KIACUC-160329-2).

## Author Contributions

DJ, YK, and HJ designed the study and provided conceptual guidance. MY and SL performed functional assays concerning CAGE-miR-140-5p-wnt1 axis. J-EL performed experiments concerning immune EM and observations of autophagosomes. DJ wrote the manuscript.

### Conflict of Interest

The authors declare that the research was conducted in the absence of any commercial or financial relationships that could be construed as a potential conflict of interest.
