# Supplementary material for: CAGE-miR-140-5p-Wnt1 Axis Regulates Autophagic Flux, Tumorigenic Potential of Mouse Colon Cancer Cells and Cellular Interactions Mediated by Exosomes
Source: Front Oncol. 2019 Nov 14;9:1240. doi: 10.3389/fonc.2019.01240 (PMC6868029; doi:10.3389/fonc.2019.01240)
Supplement: Supplementary file 1 [file Data_Sheet_1.pdf]

Supplementary Material

FIGURE S1

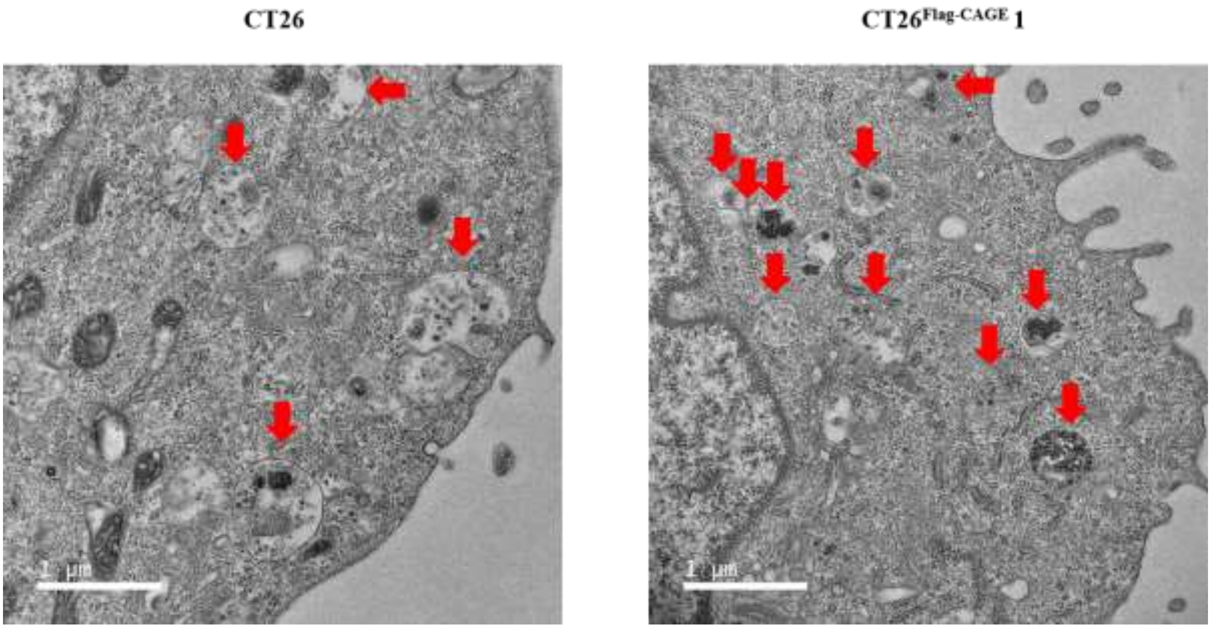

Supplementary Figure 1. CAGE enhances the formation of autophagosomes. Representative electron micrographs of CT26 cells and CT26$^{Flag\text{-}CAGE}$1 cells are shown. Arrows indicate autophagosomes.

FIGURE S2

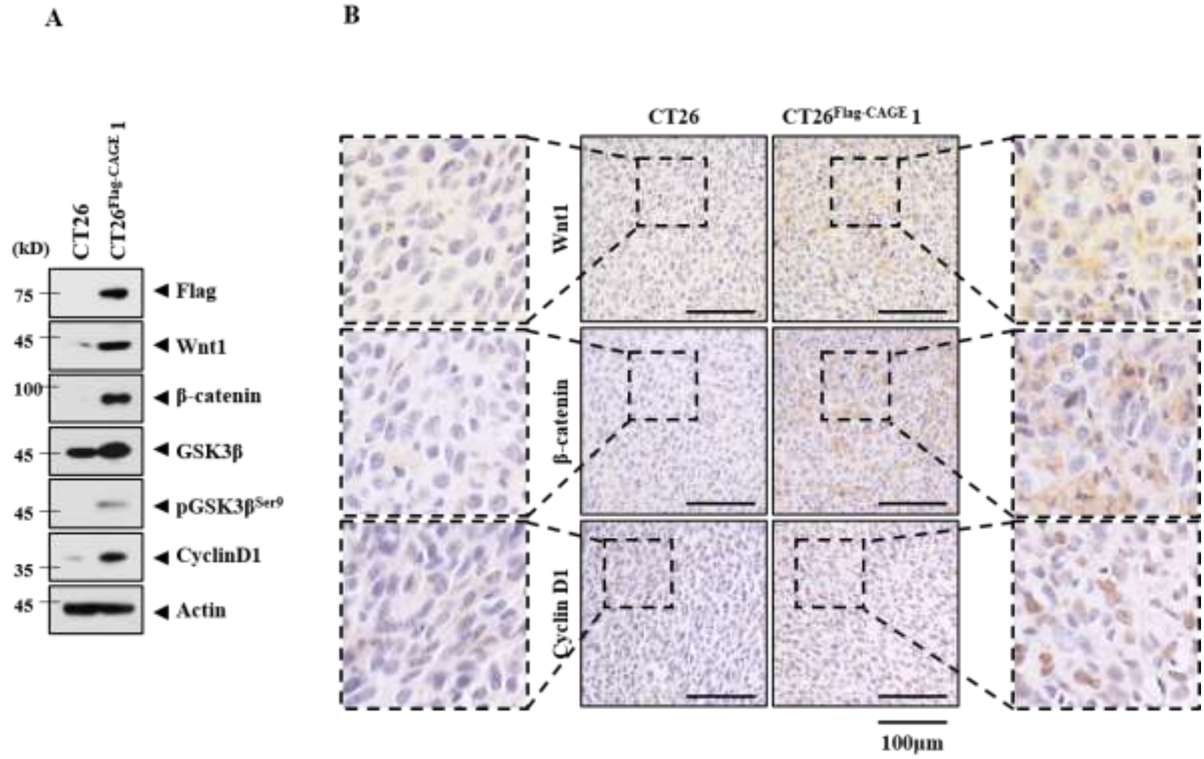

Supplementary Figure 2. The expression of Wnt1 in tumor tissues. (A) Tumor tissue lysates from the indicated cancer cells were subjected to immunoblot. (B) Immunohistochemical staining employing the indicated tumor tissues was performed.

FIGURE S3

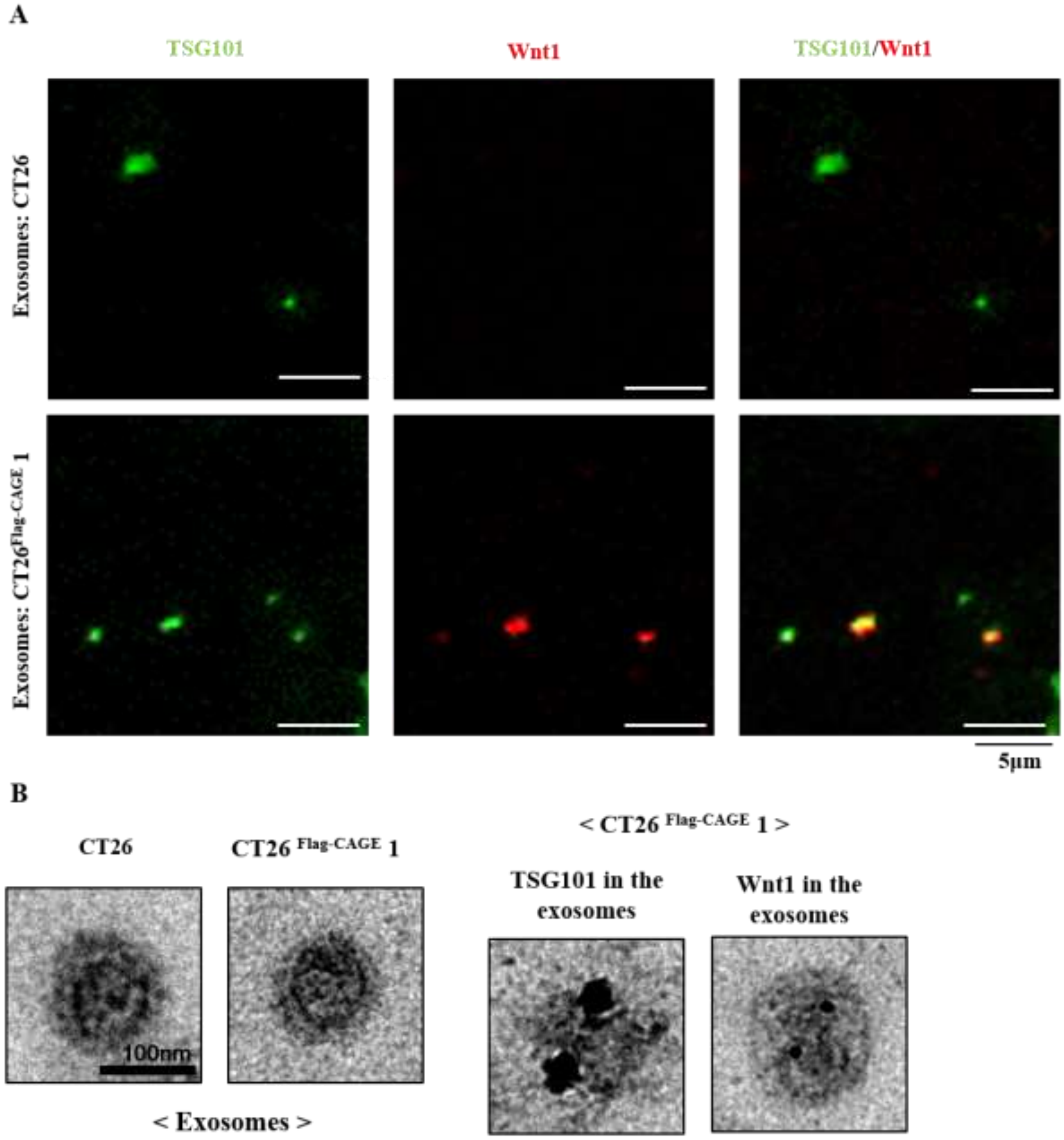

Supplementary Figure 3. Wnt1 is present within the exosomes of CT26$^{Flag\text{-}CAGE}$1 cells. (A) Exosomes isolated from the indicated cancer cells were subjected to immunofluorescence staining employing the indicated antibodies. Arrowheads indicate co-localization of Wnt1 with TSG101. (B) General appearances of isolated exosomes and immuno-gold staining images using anti-TSG101, a known membrane marker for the extracellular vesicles, and anti-Wnt1 antibodies. 25 nm and 10 nm gold particles indicate the localization of TSG101 (outer membrane of the vesicles) and Wnt1, respectively. Note that Wnt1 is shown to locate in the lumen of the vesicles.

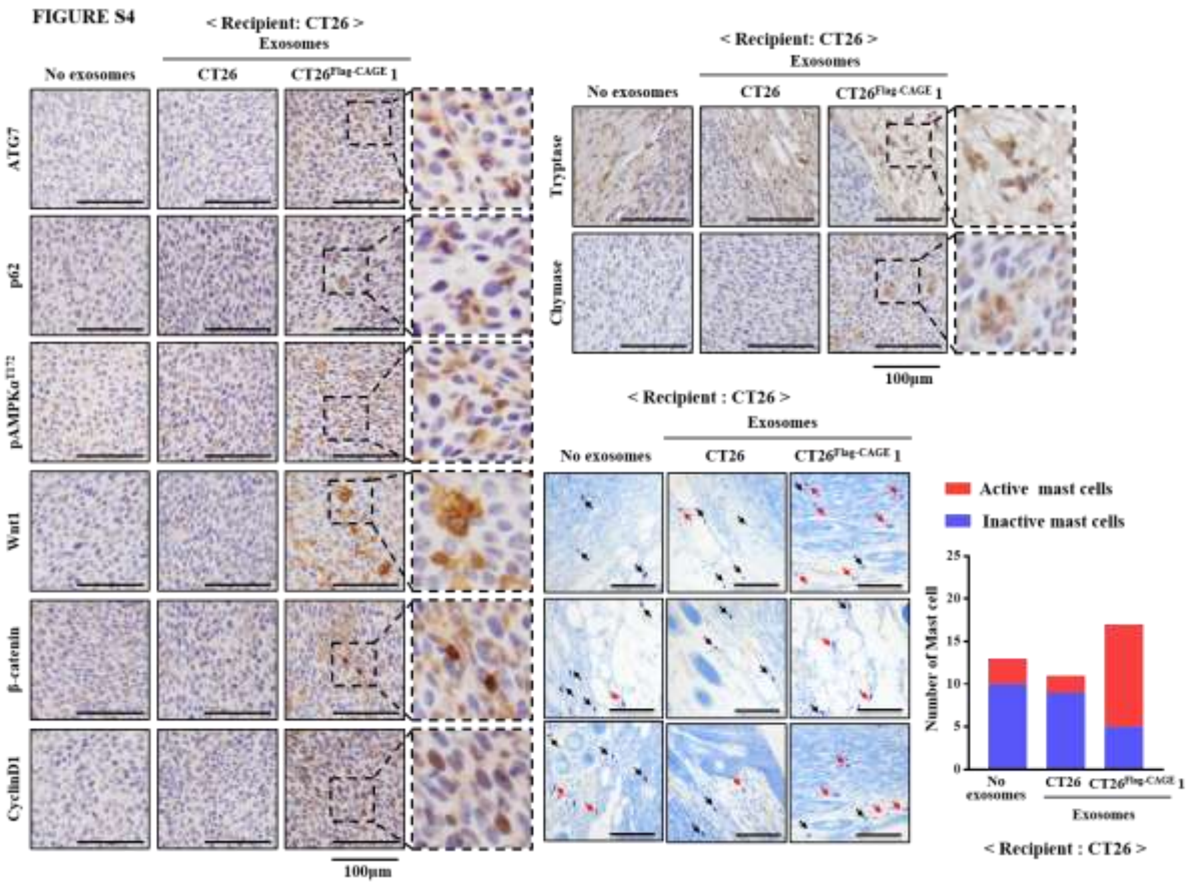

Supplementary Figure 4. CT26 cells that received exosomes from CT26$^{Flag\text{-}CAGE}$1 cells display higher expression of autophagic flux than CT26 cells that received CT26 exosomes. Immunohistochemical staining of tumor tissue employing the indicated antibody (2 μg/ml) was performed. Scale bar represents 100 μm. Tumor tissues were subjected to toluidine blue staining to determine the activation of mast cells. Red arrows represent activated mast cells.

FIGURE S5

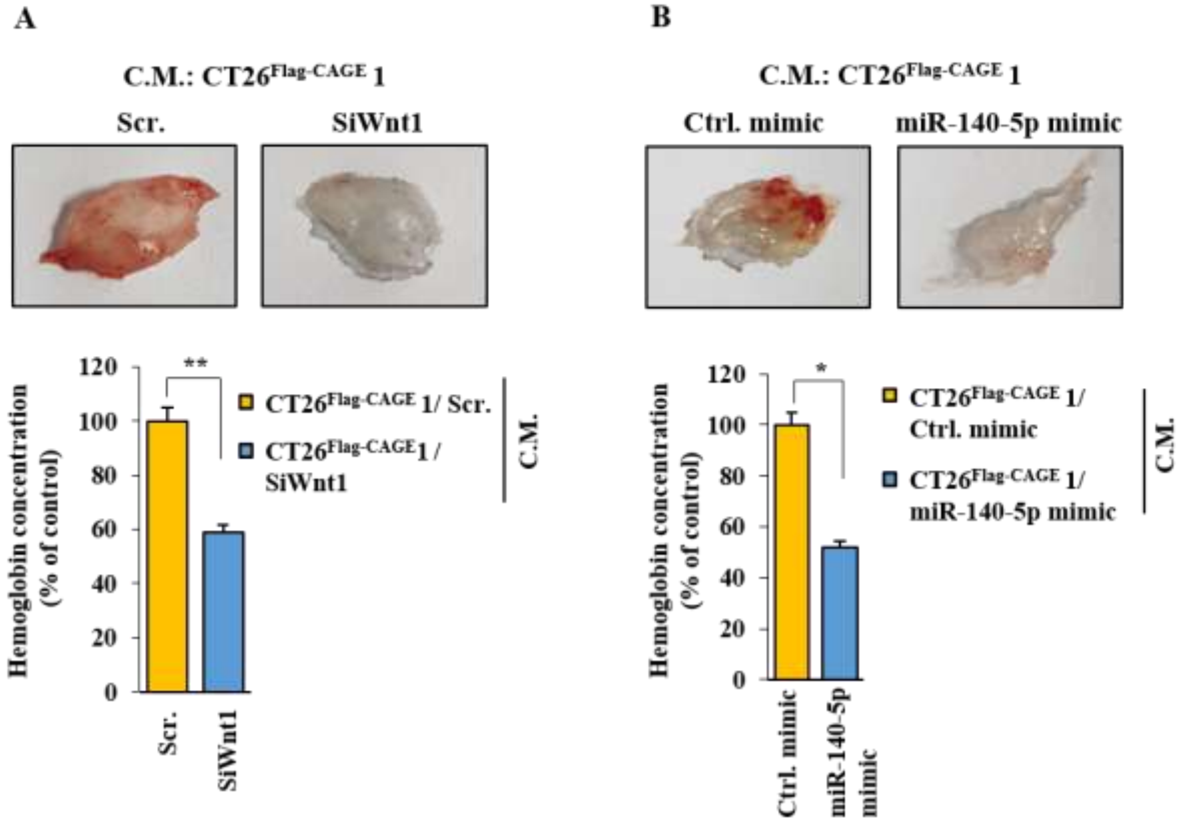

Supplementary Figure 5. Culture medium of CT26$^{Flag\text{-}CAGE}$1 cells displays angiogenic potential. The indicated cancer cells were transfected with the indicated siRNA (each at 10 nM) (A) or mimic (each at 10 nM) (B) for 48 h, followed by matrigel plug assays. *, P < 0.05; **, P <0.005.

FIGURE S6

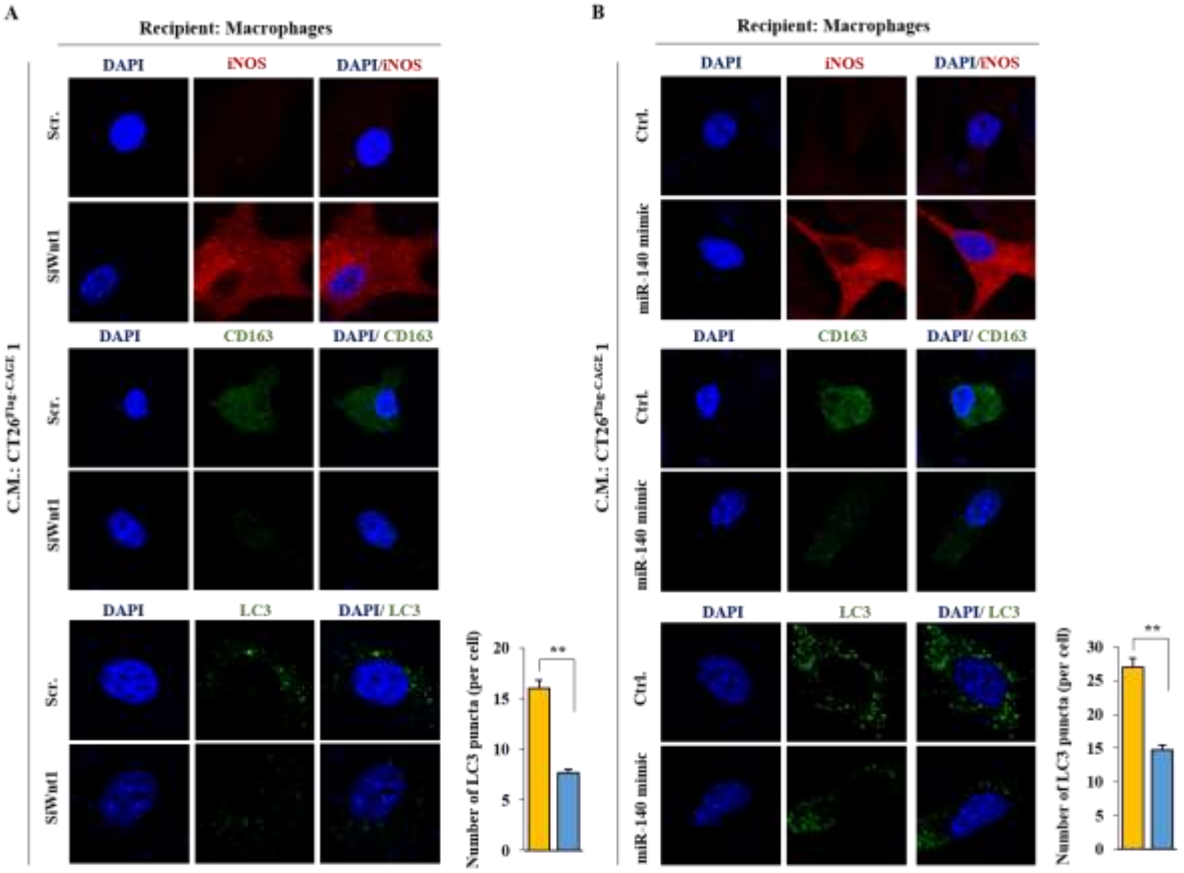

Supplementary Figure 6. Culture medium of CT26$^{Flag\text{-}CAGE}$1 cells promotes activation of macrophages. The indicated cancer cells were transfected with the indicated siRNA (A) or mimic (B). At 48 h after transfection, culture medium was added to lung macrophages. Cells were then subjected to immunofluorescence staining. **, p<0.005.

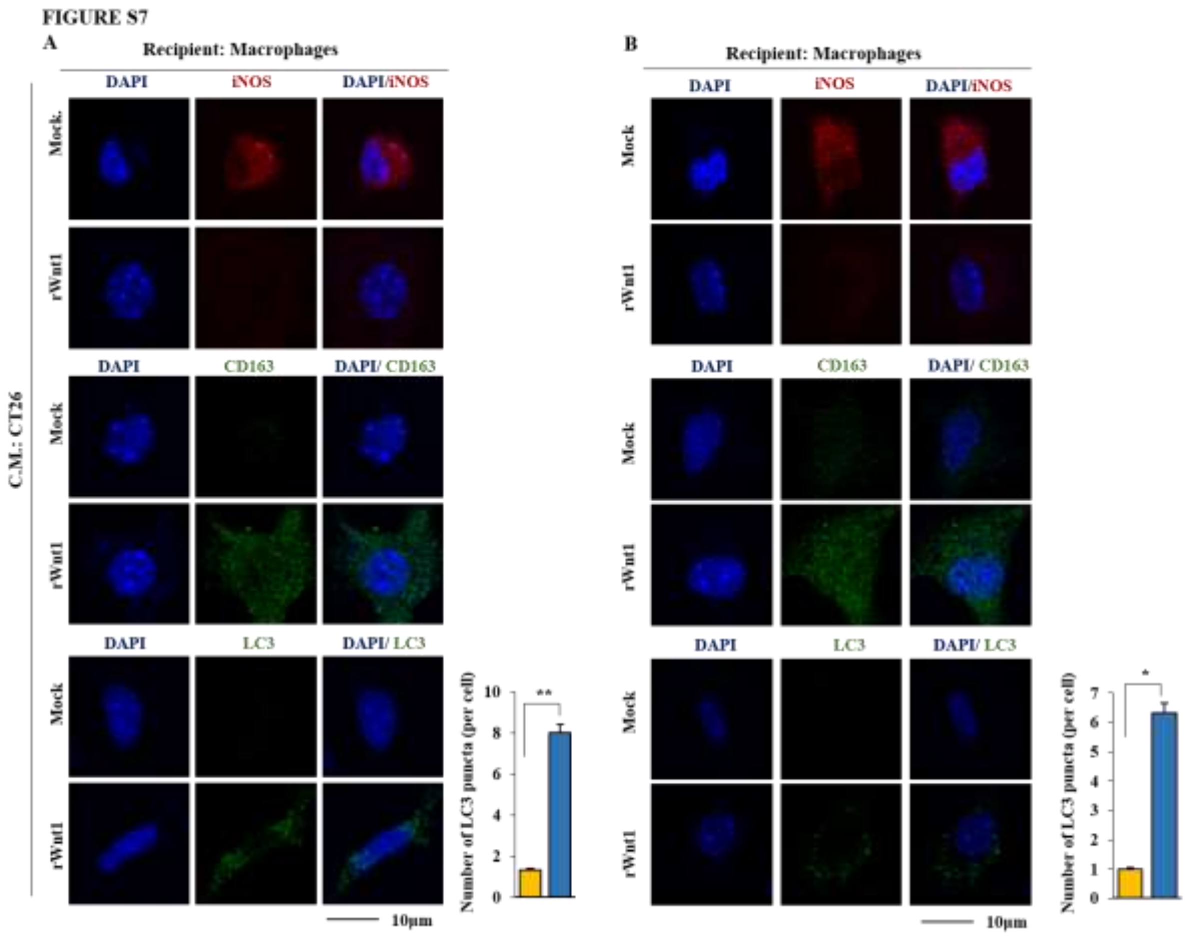

Supplementary Figure 7. Wnt1 promotes activation of macrophages by cancer cells. (A) CT26 cells were treated with recombinant Wnt1 protein (10ng/ml) for 24 h. Culture medium was then added to macrophages for 24 h, followed by immunofluorescence staining. (B) Macrophages were treated with recombinant Wnt1 protein (10ng/ml) for 24 h, followed by immunofluorescence staining. *, $P < 0.05$; **, $P <0.005$.
